# Cardiovascular morbidity, diabetes and cancer risk among children and adolescents with severe obesity

**DOI:** 10.1186/s12933-020-01052-1

**Published:** 2020-06-13

**Authors:** Cole D. Bendor, Aya Bardugo, Orit Pinhas-Hamiel, Arnon Afek, Gilad Twig

**Affiliations:** 1 Department of Military Medicine, Hebrew University of Jerusalem, Faculty of Medicine, Jerusalem, Israel; 2 Academy and Research Division, Surgeon General Headquarters, Israel Defense Forces, Medical Corps, Ramat Gan, Israel; 3 Sackler Faculty of Medicine, Tel Aviv University, Tel Aviv, Israel; 4 Institute of Endocrinology, Sheba Medical Center, Tel Hashomer, Ramat Gan, Israel; 5 Central Management, Sheba Medical Center, Tel Hashomer, Ramat Gan, Israel

**Keywords:** Severe obesity, Morbid obesity, Adolescence, Paediatrics, Youth, Cardiovascular, Diabetes, Hypertension, NAFLD, Mortality, Cancer

## Abstract

Severe obesity among children and adolescents is a significant global public health concern. The prevalence has markedly increased over the last decades, becoming common in many countries. Overwhelming rates of obesity among youth have prompted efforts to identify an evidence-based immediate- and long-term cardiometabolic risk factor profile in childhood-onset severe obesity, and to highlight gaps that require further investigation. The PubMed database was systematically searched in accordance with PRISMA guidelines. The search yielded 831 results, of which 60 fulfilled stringent criteria and were summarized in this review. The definition of severe obesity was variable, with only one half the publications using the definition BMI > 120% of the 95th percentile. Point estimates of the prevalence of at least one cardiometabolic risk factor in children with severe obesity reportedly range from 67 to 86%. Cross-sectional studies indicate that children and adolescents with severe obesity are at greater risk than those with mild obesity for type 2 diabetes, hypertension, fatty liver disease and dyslipidemia, already at childhood and adolescence. Robust epidemiological data on the long-term risk and actual point estimates in adulthood are lacking for these diseases as well as for other diseases (coronary heart disease, stroke, chronic kidney disease and cancer). Recent longitudinal studies indicate an increased risk for cardiomyopathy, heart failure, cardiovascular mortality and all-cause mortality in adulthood for adolescents with severe obesity compared to those with mild obesity. Given the alarming increase in the prevalence of severe obesity, the persistence of adiposity from childhood to adulthood and the precarious course of young adults with chronic comorbidities, the economic and clinical services burden on the healthcare system is expected to rise.

## Background

The prevalence of childhood obesity has dramatically increased over the last decades [1, 2]. This has prompted the need for obesity stratification to identify those at increased cardiometabolic risk. Substantial data have accumulated on the association of childhood obesity with cardiometabolic risk, though obesity was usually considered as a single entity [3–8]. Extreme values of body mass index (BMI), categorized as severe obesity, were once a phenomenon of adults. However, the problem is escalating in the pediatric population. According to a recent report, in many of 21 European countries, 1 in 4 children with obesity at school age have severe obesity [9]. Furthermore, 2.1% of children between ages 2 to 5 years from the US National Health and Nutrition Examination Surveys (NHANES) [10] and 1.3% of Canadians of this age group [11]; and 1.5% of toddlers (2–3 year-old) from the US National Institute (NIH) of Health Environmental Influences on Child Health Outcomes (ECHO) program [12] and 0.3% of a cohort of Canadian children aged 17 to 24 months [13] reportedly had severe obesity. Since severe obesity in childhood has become common only in the last decades, the relative contribution of this phenomenon to disease burden has only recently been approached. Identifying those with substantial risk for severe obesity and its deleterious cardiovascular sequelae is of great clinical and public health importance. This review aims to summarize (i) the association between severe obesity and the prevalence of cardiovascular risk factors, diabetes and cancer in cross-sectional studies; (ii) the relationship between severe obesity and incident cardiometabolic, cancer and mortality in longitudinal studies. We aimed to highlight gaps in evidence in order to direct future research.

## Methods

### Systematic search

A systematic search was conducted using PubMed, to identify relevant articles published through October 20, 2019. Search terms included “Obesity morbid” [MeSH] OR “severe obesity” OR “class 2 obesity” OR “class 3 obesity” AND “child*” OR “adolescen*” OR “pediat*” AND “diabetes” OR “NAFLD” OR “hypertension” OR “coronary artery disease” OR “stroke” OR “heart failure” OR “mortality” OR “cancer” OR “total cholesterol” OR “HDL” OR “LDL” OR “triglycerides” OR “blood pressure” OR “glycated hemoglobin OR “fasting glucose”. Other cardiometabolic diseases including “cardiomyopathy” OR “arrhythmias” OR “venous thromboembolism” OR “peripheral vascular disease” OR valve disorders OR “chronic kidney disease” were also systematically searched (for the detailed search strategy see Additional file 1: Table S1). Using PubMed filters, the search was limited to studies conducted on humans and published in English. We used the PRISMA checklist to ensure a high quality search and to minimize bias [14, 15].

**Fig. 1:**
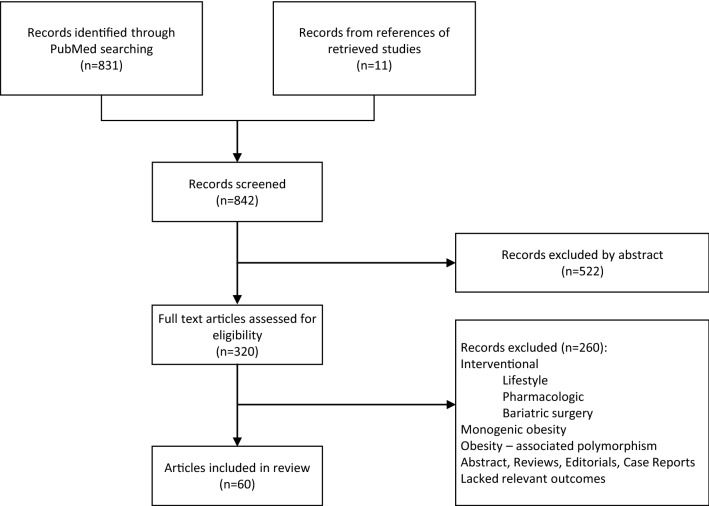
Study flowchart

Initially, 831 articles were identified. In addition to PubMed search results, we added 11 articles cited by leading studies and reviews. Manual screening by the first two authors excluded non-relevant articles, yielding 320 eligible articles. Full text articles were then reviewed. Editorials, case reports and review articles were excluded, and also studies of intervention strategy such as diet, pharmacological, or bariatric surgeries. Finally, 60 studies were included in this review (Fig. 1), of which 10 (17%) were longitudinal.

### Definitions of severe obesity

**Fig. 2:**
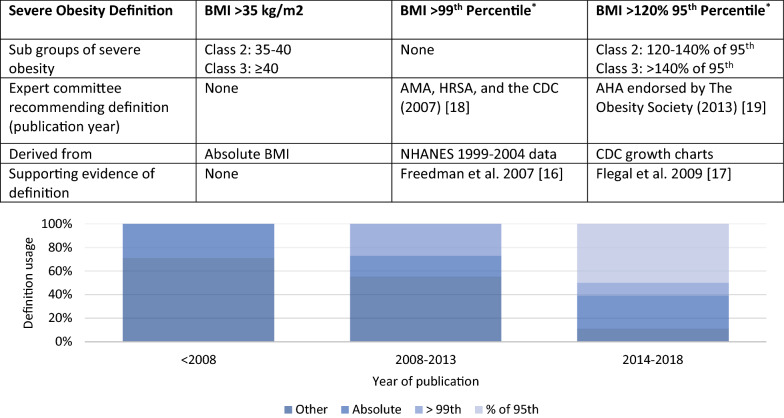
Commonly used definitions for childhood severe obesity. AMA: American Medical Association; HRSA: Health Resources and Services Administrations; CDC: Centers for Disease Control; AHA: American Heart Association; NHANES: National Health and Nutrition Examination Survey. *Age-specific and sex-specific BMI. The table shows characteristics of commonly used definitions. The chart presents the distribution of the use of the definitions by year of publication

Strategies used to define severe obesity have changed over recent years. Figure 2 outlines the common definitions [16–18] and the distribution of their use in published studies. The currently accepted definition, BMI > 120% of the 95th percentile, is derived from the Center for Disease Control (CDC) growth charts [19, 20]. This offers a flexible and intuitive means to describe and track BMI status and has been shown to better discriminate cardiometabolic risk [17]. However, since its establishment in 2013, only one half the articles published thereafter utilized this definition. Other classifications of severe obesity according to absolute BMI cut-off, z-score, and ≥ 99th percentile are commonly used, despite disputed adiposity estimation [21, 22]. The inclusion of studies in this review was not limited to a specific definition of severe obesity. Some of the studies further classified severe obesity into class 2 obesity and class 3 obesity.

## Results

### Sex and ethnicity representation

Overall, females represented only about 20% of the children and adolescents in the 60 studies reviewed (Fig. 1). This proportion increased when large population-based cohorts that were based mostly on male conscripts [23–25] were excluded. While most studies did not assess outcome-related sex differences, some showed comparable rates of cardiometabolic risk factors among girls and boys [2, 16, 26, 27]. Others indicated higher risk in boys, with greater risk for abnormal systolic blood pressure, TGs and glycated hemoglobin [28–30]. Of note, evidence from large scale studies that analyzed obesity as a single entity showed that at a given BMI, risks for incident diabetes [31], and cardiovascular [32] and other cause-specific mortality [33], were higher in females than in males.

Most US cohorts were based on national representative data or included representative proportions of ethnic minorities, including African Americans and Hispanics [26, 28, 34, 35]. Whereas only few studies have reported similar prevalence for abnormal cardiometabolic risk factors [36], most have shown higher risk among African Americans and Hispanic populations [37–39]. Among American adolescents, the steepest rise in hypertension prevalence was detected among Hispanics, and the least among African Americans [40]. In contrast, a recent nationwide study showed that descendants of African immigrants to Israel had higher risk for hypertension at a given BMI level regardless of their socioeconomic background, and that this effect was more pronounced at very high BMI values [41]. Cohorts outside the US showed higher risk among immigrant than native populations [42]. A recent report from the International Childhood Cardiovascular Cohort (i3C) group cited female sex and black ethnicity as factors that increase the probability of children remaining with severe obesity as adults [43].

### Cardiometabolic risk factors

**Fig. 3:**
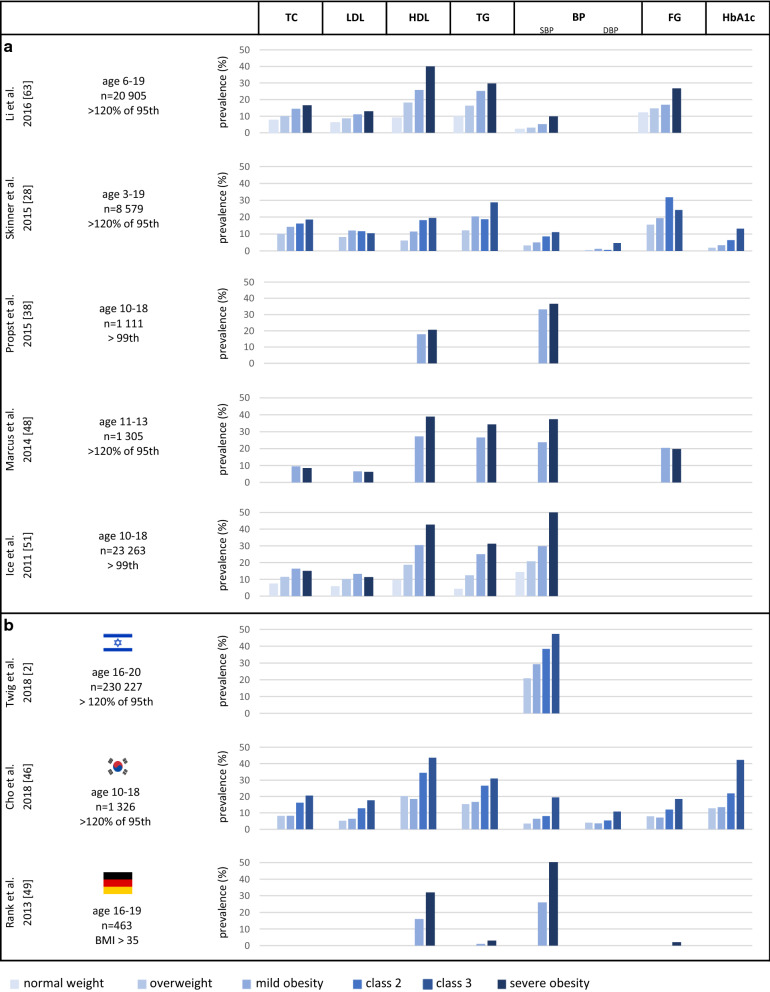
Prevalence of abnormal cardiometabolic risk factors by BMI group in cross-sectional studies. TC: total cholesterol; LDL: low-density lipoprotein cholesterol; HDL: high-density lipoprotein cholesterol; TG: triglycerides; BP: blood pressure; SBP: systolic blood pressure; DBP: diastolic blood pressure; FG: Fasting Glucose; HbA1c: haemoglobin A1c. A single panel under BP represents abnormal values of SBP or DBP. Abnormal values of fasting plasma glucose (≥ 100 mg/dl) and glycated hemoglobin (> 5.7%) are those recommended by the American Diabetes Association for identifying persons at high risk for diabetes (at least in the prediabetes range). Studies based on cohorts from the United States (a) and other countries (b) are presented. The definition of severe obesity varied between studies, as did the thresholds that defined abnormal values of cardiometabolic morbidity (Additional file 1: Table S2)

Substantial data have accumulated in recent years on the cross-sectional association of childhood severe obesity with immediate cardiometabolic risk factors. Most studies examined lipid profiles (total cholesterol, triglycerides (TG), low-density lipoprotein (LDL), high-density lipoproteins (HDL)) blood pressure (systolic BP and diastolic BP) and glycemic status (fasting glucose and less often glycated hemoglobin). Point estimates of prevalences of abnormal values of these risk factors by BMI group are summarized in Fig. 3. While metabolic syndrome was suggested to be more prevalent in children with severe than mild obesity [42, 44, 45], only few studies focused on elucidating whether severe obesity carries an increased cardiometabolic risk compared with mild obesity [26–28, 38, 45–49] (Table 1). Of note, definitions of abnormal values varied considerably between studies (Additional file 1: Table S2), and the point estimates of the prevalence of at least one cardiometabolic risk factor in children with severe obesity ranged from 67 to 86% [16, 45, 50–52].

**Table 1:** Cardiometabolic risk factors among children with severe compared to mild obesity in cross-sectional studies

Article	Age range		Cardiometabolic risk factors												
			Total Cholesterol		LDL		HDL		Triglycerides		Systolic BP	Diastolic BP	Fasting Glucose		HbA1c
Cho et al. [46] (n = 1 328)	10–18	Mean values	Mild: 163.9 (160.9–166.8) Class 2: 171.6 (167.5–175.6)		Mild: 93.6 (90.9–96.4) Class 2: 100.7 (97.1–104.3)		Mild: 48.6 (47.6–49.6) Class 2: 46.0 (44.6–47.4)		Mild: 94.3 (88.5–100.4) Class 2: 109.0 (102.3–116.0)		NS	NS	NS		NS
Zhang et al. [26] (n = 44 630)	7–18	Prevalence of abnormal values									Boys: mild: 39.93% Class 3: 50.54% Girls: Mild: 39.53% Class 3: 53.66%				
Skinner et al. [28] (n = 8 579)	3–19	Mean values*	Mild: 167.2 (165.3–169.1) Class 2: 166.0 (163.0–169.0) Class 3: 169.6 (163.4–175.9)		NS		Mild: 46.7 (46.1–47.3) Class 2: 43.5 (42.6–44.5) Class 3: 41.3 (40.2–42.4)		Mild: 113.2 (103.5–123.0) Class 2: 113.3 (103.3–123.3) Class 3: 143.2 (113.6–172.8)		Mild: 111.0 (110.4–111.7) Class 2: 112.6 (111.6–113.7) Class 3: 116.2 (114.6–117.8)	Mild: 58.8 (57.8–59.9) Class 2: 58.7 (57.4–60.1) Class 3: 64.5 (62.7–66.4)	Mild:95.1 (93.8–96.3) Class 2: 96.7 (94.6–98.7) Class 3: 96.5 (94.0–98.9)		Mild: 5.20 (5.17–5.24) Class 2: 5.30 (5.25–5.35) Class 3: 5.37 (5.29–5.44)
		Prevalence of abnormal values*	Mild: 14.27 (12.46–16.07) Class 2: 16.19 (12.35–20.03) Class 3: 18.59 (12.86–24.32)		NS		Mild: 11.40 (9.84–12.97) Class 2: 18.18 (14.35–22.00) Class 3: 19.53 (13.94–25.12)		Mild: 20.35 (16.48–24.22) Class 2: 18.81 (12.76–24.86) Class 3: 28.82 (18.22–39.42)		Mild: 5.02 (3.87–6.17) Class 2: 8.52 (5.76–11.27) Class 3: 11.10 (6.10–16.09)	Mild: 1.20 (0.47–1.94) Class 2: 0.60 (0.16–1.37) Class 3: 4.66 (1.92–7.39)	Mild: 19.42 (14.32–24.52) Class 2: 31.77 (23.90–39.65) Class 3: 24.27 (14.54–34.00)		Mild: 3.40 (2.26–4.53) Class 2: 6.38 (4.02–8.73) Class 3: 13.19 (8.07–18.30)
		RR	NS		NS		Class 2: 1.65 (1.31–2.01) Class 3: 1.89 (1.35–2.66)		Class 2: NS Class 3: 1.63 (1.08–2.47)		Class 2: NS Class 3: 2.24 (1.42–3.54)	Class 2: NS Class 3: 4.57 (1.88–11.06)	Class 2: 1.67 (1.26–2.22) Class 3: 1.24 (0.78–1.96)		Class 2: NS Class 3: 2.59 (1.55–4.34)
Propst et al. [38] (n = 1 111)	12.7 ± 3.4	Mean Values	NS		NS		Mild: 44.9 ± 14.0 Severe: 43.1 ± 11.9				NS		NS		
		Prevalence of abnormal values					NS				NS				
Shah et al. [47] (n = 447)	10–24	Mean Values	NS		NS		NS		NS		Mild: 117 ± 11 severe: 121 ± 12	NS	NS		NS
Marcus et al. [48] (n = 1 305)	~11 (6th grade)	Mean Values	NS		NS		Mild: 47.1 ± 9.9 severe: 43.8 ± 9.3		Mild: 108.8 ± 59.3 severe: 125.9 ± 70.9		Mild: 110.2 ± 9.7 severe: 112.0 ± 11.1		Mild: 66.0 ± 7.8 severe: 70.4 ± 8.6		NS
		Prevalence of abnormal values	NS		NS		Mild: 27.2% Severe: 38.9%		Mild: 26.6% Severe: 34.3%		Mild: 23.8% Severe: 37.4%		NS		
Rank et al. [49] (n = 43)	6–19	Mean Values	NS		Boys: Mild: 98.8 ± 32.7 Severe: 110.0 ± 36.5	Girls: NS	Boys: Mild: 55.1 ± 12.9 Severe: 44.2 ± 10.4	Girls: Mild: 53.1 ± 13.3 Severe: 47.0 ± 10.5	Boys: Mild: 53.7 ± 27.4 Severe: 71.9 ± 31.3	Girls: Mild: 59.8 ± 20.9 Severe: 77.0 ± 29.7	Boys: Mild: 124 ± 11 severe: 135 ± 14	Girls: Mild: 122 ± 8 Severe: 128 ± 10	Boys: Mild: 78 ± 7 Severe: 84 ± 9	Girls: Mild: 77 ± 6 Severe: 82 ± 7	NS
		Prevalence of abnormal values					Mild: 16% Severe: 32%		NS		Mild: 26% Severe: 58%		NS		
Calcaterra et al. [45] (n = 191)	11.15 ± 3.4	OR**	Metabolic syndrome**: 3.3 (1.6–7.0)												
Boyd et al. [27] (n = 497)	2–18	Prevalence of abnormal values	Boys: Mild: 32.1 Severe: 15.4	Girls: NS	Boys: Mild: 25.9 Severe: 12.2	Girls: NS	Boys: Mild: 29.3 Severe: 40.3	Girls: NS	Boys: NS	Girls: Mild: 24.1 Severe: 11.1					

LDL: low-density lipoprotein cholesterol; HDL: high-density lipoprotein cholesterol; BP: blood pressure; HbA1c: hemoglobin A1c; NS: not statistically significant; RR: relative risk; OR: odds ratio

Total cholesterol, LDL, HDL, triglycerides and fasting glucose are given in mg/dl values. Systolic and diastolic blood pressure are given in mmHg values. HbA1c is given in % units. Presented are mean values, prevalences and risk ratios for different cardiometabolic risk factors. Shown are only articles that statistically compared cardiometabolic risk factors in severe obesity with those in mild obesity. The definition of severe obesity varied between the studies. Class 2 obesity and class 3 obesity are used to further stratify obesity severity. Different methods and cut-off points were used to define abnormal values of cardiometabolic risk factors. These are detailed in Additional file 1: Table S2. For articles that did not report the age range, the mean age ± standard deviation is presented. Plus-minus values are means ± standard deviations. Numbers in parenthesis represent 95% confidence intervals

* P-values were calculated for trends across weight categories. ** Metabolic syndrome was determined as three and above of the following: (1) BMI > 97th percentile, (2) Triglycerides>95th percentile, (3) HDL<5th percentile, (4) Systolic BP OR Diastolic BP> 95th percentile, (5) Impaired glucose tolerance

### Dyslipidemia

Dyslipidemia that is associated with obesity is typically characterized by increased TG and free fatty acids, decreased HDL levels and a normal or slightly increased LDL level. Most studies that assessed cardiometabolic risk factors according to BMI status reported higher mean values of TG, and lower mean values for HDL [53–62] (Table 1); all were cross-sectional. Higher prevalences [28, 38, 46, 48, 49, 51, 63] and increasing trends of abnormal values were observed with increasing BMI categories (Fig. 3). In most of the studies, LDL was not significantly elevated in severe obesity compared to class I obesity.

### Hypertension

Point estimates on the prevalence of hypertension in children are mainly based on a single measurement of elevated blood pressure; this was shown to overestimate disease rates [64]. Prevalences of well-defined hypertension among children with severe obesity were fairly consistent between studies, and ranged from 9 to 17% [28, 39, 65, 66]. In a cross-sectional retrospective US cohort, children with severe obesity and mild obesity had an odds ratio of 4 and 2 for hypertension, respectively, compared to children with normal weight [67]. In a nationwide cross-sectional Israeli cohort, odds ratios for hypertension ranged from 2.1 to 3.4 among girls and boys with severe obesity, and from 1.4 to 1.8 among those with mild obesity, compared to children with overweight [2]. None of the studies included in the current review assessed the future risk in a longitudinal manner for adult hypertension among children and adolescents with severe obesity. Notably, data on tracking of blood pressure from childhood to adulthood demonstrated a strong correlation and a higher prevalence of youth-onset hypertension in children with high blood pressure [64, 68, 69].

### Prediabetes and type 2 diabetes

In parallel to the major rise in pediatric obesity, incidences of youth prediabetes and type 2 diabetes have increased significantly in recent years [7, 70, 71]. Prediabetes was detected in 22–36% of severely obese children and adolescents in cross-sectional studies [4, 38, 72]. A retrospective single center study indicated a greater prevalence of prediabetes (assessed by glycated hemoglobin of 5.7–6.4%) in children and adolescents with severe obesity compared to their peers with mild obesity (27.3% vs. 19.5%, respectively; *P* < 0.001) [38]. An NHANES-based cross-sectional US study reported a 1.7 risk ratio (95% CI 1.3–2.2) for impaired fasting plasma glucose (≥ 100 mg/dl) among children and adolescents with class 2 obesity compared to those with mild obesity [28].

The most prominent clinical risk factor for type 2 diabetes in children and adolescents appears to be severe obesity. The average BMI of children with type 2 diabetes in published reports ranges from 35 to 39 kg/m^2^; about one-third of children with type 2 diabetes were found to have a BMI greater than 40 kg/m^2^ and 17% a BMI greater than 45 kg/m^2^ [73]. In a nationwide, cross-sectional study of adolescents, the prevalence of diabetes was 1.5% among those with severe obesity. Moreover, statistically significant rises in odds ratios for diabetes were reported for adolescents with class 2 and class 3 obesity (19.1 [95% CI 12.3–29.6] and 38.0 [95% CI 22.6–64.0], respectively) compared to mild obesity (5.59 [95% CI, 3.66–8.54]); overweight was the reference group [2]. In a two-year longitudinal follow-up of children with obesity, 8 of 117 developed type 2 diabetes; severe obesity was the strongest predictor of incident diabetes [74].

Children as young as age 3.5 years were documented with type 2 diabetes. These children were all with severe obesity [75, 76]. Importantly, if pediatric type 2 diabetes mirrors the adult experience, many affected individuals may go undiagnosed. This is supported by the findings of a specialized obesity clinic, wherein previously undiagnosed type 2 diabetes was identified in 4% of adolescents with obesity [72]. Microvascular complications of type 2 diabetes have been identified at an early age and are often present at diagnosis [77]. Finally, data show that youth with type 2 diabetes are at risk for earlier onset and more aggressive progression of diabetes-related complications than adults with type 2 diabetes, and than youth with type 1 diabetes [78, 79]. The risk is greater in those with young-onset vs. later-onset type 2 diabetes. Furthermore, a significant mortality excess was noted in youth with type 2 diabetes, with a twofold increased hazard for death, which occurred after a shorter disease duration and at a relatively younger age [78].

### Fatty liver disease

Fatty liver disease is strongly associated with obesity, and nonalcoholic fatty liver disease (NAFLD) became the most common childhood chronic liver disease in many Western countries [80]. NAFLD prevalence was estimated as 40–60%, based on non-invasive imaging in cross-sectional cohorts of children with severe obesity [81–83]. The prevalence of NAFLD has been reported to increase in parallel with increasing severity of obesity [80, 84].

The paradigm and rates of progression from NAFLD, through nonalcoholic steatohepatitis (NASH) to cirrhosis and hepatocellular carcinoma, has not been thoroughly studied in the pediatric population. Though biopsy is the gold standard for the diagnosis of NASH, only a few pediatric studies were based on histopathologic data [85–87]. The available data indicate unique features of liver inflammation and fibrosis, compared to the classic pattern in the adult population. Compared to adults with matched BMI and metabolic risk profile, adolescents with severe obesity were shown to have more advanced liver disease, characterized by higher prevalence of definitive NASH (62% vs. 25%, *P *= 0.009) and fibrosis (83% vs. 29%, *P *= 0.002), and also higher systemic inflammatory markers [87]. Others have shown a distinct histopathological pattern dominated by portal inflammation and portal fibrosis; albeit scarce lobular inflammation, hepatocyte ballooning and perisinusoidal fibrosis [86]. Thus, the natural history of the disease and its future consequences are uncertain. Of note, pediatric fatty liver disease has been shown to cluster with cardiometabolic risk factors. This is exemplified by the presence of type 2 diabetes in 10% of adolescents with biopsy proven NAFLD [88, 89]. Fatty liver may prove to be a mediator of the metabolic syndrome, by dysregulation of both glucose and lipid metabolism. The metabolic syndrome was reported in 50% of children with obesity and NAFLD, compared to 15% in a control group without NAFLD, matched for sex, age and BMI [88].

### Heart failure and cardiomyopathy

**Fig. 4:**
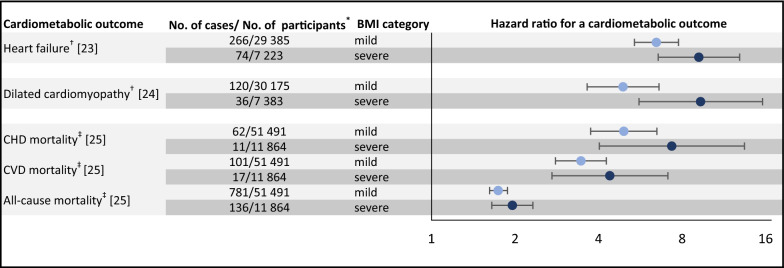
Cardiometabolic outcomes in adulthood of adolescents with severe versus mild obesity in national longitudinal cohorts. CHD: coronary heart disease; CVD: cardiovascular disease. Severe obesity and mild obesity were defined as BMI ≥ 35 and 30 ≤ BMI < 35 kg/m^2^, respectively. Cox proportional hazard models were used in all the studies. The horizontal axis is presented in the logarithmic scale. * The presented numbers of cases and participants were derived from the unadjusted models. †Swedish national cohort. Heart failure was determined as the first event of heart failure hospitalization. The reference groups were 18.5 < BMI < 20.0 kg/m^2^. Hazard ratios (HRs) were adjusted for age at entrance to the study, the year of entrance to the study, test center, comorbidities at baseline, parental education, systolic and diastolic blood pressure, muscle strength and fitness. HRs for heart failure were also adjusted for IQ level. HRs for cardiomyopathy were also adjusted for alcohol and substance use disorder. ‡Israeli national cohort. The reference group was 17.5 < BMI < 20.0 kg/m^2^. HRs were adjusted for age, birth year, sex, socioeconomic status, country of origin, education level and height

Two historical longitudinal cohort studies based on data from the Swedish Conscript Registry showed an association of severe obesity in adolescence with heart failure in adulthood. Among male adolescents with severe obesity (BMI ≥ 35 kg/m^2^), a hazard ratio of 9.2 for hospitalization from heart failure was reported, compared to their peers with normal BMI, with a follow-up of 23.0 years [23] (Fig. 4). A change in BMI during puberty was associated with an up to twofold increased risk for heart failure [90]. A recent longitudinal study from this nationwide cohort showed a strong association of adolescent BMI with elevated risk of cardiomyopathy in adulthood, especially dilated cardiomyopathy. Point estimates increased with higher BMI categories, and were above eightfold in adolescents with BMI ≥ 35 kg/m^2^ compared to those with normal BMI [24] (Fig. 4).

### Coronary heart disease, stroke, other cardiovascular disease and chronic kidney disease

The systematic search described herein did not identify cross-sectional or longitudinal articles that assessed the association between severe obesity in childhood and incident coronary artery disease, stroke, other cardiovascular disease and chronic kidney disease (see Additional file 1: Table S1 for the detailed search strategy).

### Cancer

A recent longitudinal study of Israeli adolescents who were evaluated during 1967–2010 and were followed for 20 years demonstrated that increased BMI at adolescence is associated with an increased incidence of cancer. The hazard ratios for cancer were about 1.26–1.27 for adolescents with obesity, though obesity was treated as a whole without subdivisions to mild and severe obesity [91]. The association that was accentuated in the late period of the cohort (1982–1997) could be explained by the increase in the fraction of adolescents with severe obesity in the study period [2]. The systematic search did not identify any epidemiological studies that have assessed the link between severe obesity in childhood and incident cancer. Genome-wide methylation analysis identifies specific epigenetic marks in children with severe obesity that were not found in those with mild obesity and were associated with “IRS1 target genes” pathway and different cancer traits including pancreatic cancer, breast tumors, hepatocellular carcinoma and colon cancer [92].

### Mortality

An Israeli national longitudinal study that followed 2.3 million male and female conscripts for up to 18.4 years showed an association of severe obesity at adolescence with mortality at adulthood [25]. The hazard ratios for fatal coronary events were 7.1 (3.9–13.0) vs. 4.8 (3.7–6.4) among those with BMI higher than 35 kg/m^2^ compared to those with BMI of 30–35 kg/m^2^. The point estimates for total cardiovascular mortality were 4.3 (2.7–6.9) vs. 3.4 (2.8–4.2), and for all-cause mortality 2.0 (1.7–2.3) vs. 1.7 (1.6–1.9) [25] (Fig. 4). The current systematic search did not identify other studies that investigated mortality as an outcome of severe obesity.

## Discussion

During the last two decades, the prevalence of severe obesity in children has increased by twofold both in the US [93–95], and in other countries [2, 96]. In this systematic review of the association of childhood severe obesity with immediate cardiometabolic risk factors, we focused on the risk of severe compared to mild obesity. The data show that children with severe obesity are at greater risk for dyslipidemia, hypertension, type 2 diabetes and fatty liver disease than children with mild obesity. However, the long-term risk and actual point estimates in adulthood are lacking.

Several studies have reported associations of severe obesity in children with increased prevalence of cardiovascular risk factors. Data of children and adolescents from the US NHANES showed greater prevalence of abnormal TGs, diastolic blood pressure and glycated haemoglobin levels in class 3 than class 2 obesity [28]. In addition, significantly higher prevalences of abnormal HDL, systolic blood pressure and glycated hemoglobin levels were observed in class 2 than in mild obesity. The magnitude of associations of severe obesity with cardiovascular risk factors in children differs by the specific risk factor [38, 47, 48, 51]. Notably, the latent period for developing significant cardiometabolic morbidity may require several decades [24, 25]. Therefore, the current data evidently does not reflect the actual morbidity, given the sharp increase in severe adolescent obesity in some Western countries over the recent period [2, 93, 94].

Type 2 diabetes risk is predominantly affected by obesity class. Higher BMI has been found to be associated with younger age at diabetes diagnosis [97, 98]. Young onset diabetes has a greater productivity burden and was associated with higher rates of cardiovascular morbidity and mortality [99–101]. Thus, the relative contribution of childhood severe obesity to type 2 diabetes is of high clinical and public health importance. A 1.7-fold (95% CI 1.3–2.2) increased risk for dysglycemia (in the prediabetes or diabetes range) was reported among children with class 2 obesity compared to those with mild obesity [28]. Numerous studies have reported a strong relation between obesity in youth and subsequent type 2 diabetes in adulthood, but none compared outcomes among those with mild and severe obesity [31, 102–110].

Sociodemographic variables are potential confounders of the association between degree of childhood obesity and cardiometabolic risk. Although some analyses were adjusted for sex and ethnicity, most studies did not adjust the observed risk for socioeconomic confounders that have been shown to be closely associated with both severe obesity and cardiometabolic morbidity [111]. However, in a cross-sectional study of pre-recruitment adolescents, the odds ratios for type 2 diabetes and for hypertension among individuals with mild compared to severe obesity were not materially affected by adjustment for residential socioeconomic status based on locality of residence and for education level assessed by years of formal schooling [2]. A recent Australian study exemplifies the complexity of sociodemographic adjustment [112]. Among children with severe obesity, average neighborhood education/occupation and family education level were negatively associated with BMI, waist circumference and body fat percentage, but not with cardiometabolic risk factors. Neighborhood walkability in that study was related to lower waist circumference. However, better access to basic shopping facilities including playgrounds and parks was also related to higher prevalences of dyslipidemia and fatty liver. A study from New Zealand revealed that severe obesity was more common among adolescents living in areas of high deprivation [113]. Household dysfunction has been associated with severe obesity. Furthermore, traumatic life experiences, such as physical and sexual abuse during childhood and adolescence, have been far more common in adults with severe obesity [114] and also in children [115].

Children with early-onset severe obesity display more pronounced obesogenic behaviours than do their peers with overweight or mild obesity [116]. Among the factors that have been found to be associated with severe obesity among young children are: lower consumption of fruits and vegetables, higher consumption of fast food, less outdoor play, shorter sleep duration, lack of bedtime rules, increased screen time and less involvement in team sports [117, 118]. Thirty percent of adolescents with severe obesity were current cigarette users, compared with 14% among those with healthy weight students [113]. These data on modifiable cardiometabolic risk factors were not considered by most of the studies included in the current review. In 2016, the US NIH initiated the ECHO program in an attempt to address environmental origins of childhood obesity [12]. Data generated from this initiative should assist in setting the stage for intervention studies aimed to lessen the burden of childhood obesity. Clinicians should promote healthier diet, physical activity, and avoidance of smoking, particularly among adolescents with obesity [119], to mitigate cardiometabolic risk.

The above data suggest that cardiovascular disease and risk factors are intensified in severe compared to mild obesity, and appear at earlier ages. The existence of morbidity related to severe obesity already in childhood or youth is especially detrimental since it affects this young population in the most productive years of adulthood. Two pieces of data should be considered in this instance. First, severe obesity has increased among US children, even as young as 2–5 years old [1]. Second, youth and young adults may be less aware of cardiometabolic morbidities such as type 2 diabetes than older adults [120]. The upshot is delayed diagnosis and worse clinical course. This is exemplified by the existence of complications already at diagnosis and the harmful course of the disease [78]. Notably, mortality among individuals with diabetes was shown to decline in all age groups except young adults (ages 20 to 44 years) [121]. Estimated diabetes costs in the US in 2017 were $327 billion, which includes $237 billion in direct medical costs and $90 billion in lost productivity. Annual per capita health care expenditure is 2.3 times higher for people with than without diabetes. A large portion of medical costs associated with diabetes costs is for comorbidities [122], which were much more frequently reported in young individuals with diabetes [123]. Two recent trends are of particular concern: the decreasing age for the onset of severe obesity among preschool children and the decreasing age for the onset of type 2 diabetes in youth. These, together with their deleterious clinical courses, likely portend a pronounced increase in economic burden over the ensuing years.

This systematic review has a number of limitations. As noted above, studies that have addressed the risk for cardiometabolic complications in children and adolescents have used highly variable definitions of severe obesity; less than half of them used BMI > 120% of the 95th percentile, which is recommended. Since this definition has a discriminatory advantage in identifying children with severe obesity who are at increased cardiometabolic risk, point estimates may be underestimated [22]. Furthermore, point estimates are susceptible to age and sex bias, although this may be mitigated by adjustment for these variables. This caveat should be particularly emphasized in longitudinal studies that used definitions that were shown to have weaker correlations with sequential obesity measurements [21]. Second, our systematic search was based on an explicit definition of severe or morbid obesity. Therefore, studies that did not use this terminology but included data regarding higher classes of obesity may have been missed. We expect that the number of such studies is low, and we acknowledged some of them in this review. Third, the assessment of sex-based differences is limited, given the low disproportionate representation of females and the minority of the studies that stratified the analyses by sex. Despite the above limitations, important observations are evident regarding the burden of comorbidities in children with severe obesity at an early age.

## Conclusion

In summary, significant cardiovascular morbidity and higher risk of all-cause mortality have been reported in children and adolescents with severe obesity. The alarming increase in the prevalence of severe obesity, specifically among very young children, is likely to pose major challenges for the future burden of cardiometabolic disease. Longitudinal studies with follow-up into adulthood are needed to characterize populations that should be specifically targeted for prevention and early intervention. Accordingly, sex-, ethnic- and other sociodemographic-specific mediators should be delineated. Given the precarious course of young adults with chronic comorbidities such as diabetes, the clinical, financial and public health burden attributed to severe obesity is expected to climb.

## Supplementary information

**Additional file 1: Table S1.** Search terms used in the systematic search. **Table S2.** Methods and cut-off points that were used to define abnormal values of cardiometabolic risk factors.

## Data Availability

Data sharing is not applicable to this article as no datasets were generated or analysed during the current study.

## Abbreviations

BMI: Body mass index
CDC: Center for Disease Control
TG: Triglycerides
LDL: Low-density lipoprotein
HDL: High-density lipoprotein
BP: Blood pressure
NAFLD: Nonalcoholic fatty liver disease
NASH: Nonalcoholic steatohepatitis
