# Supplementary material for: Cardiovascular morbidity, diabetes and cancer risk among children and adolescents with severe obesity
Source: Cardiovasc Diabetol. 2020 Jun 13;19:79. doi: 10.1186/s12933-020-01052-1 (PMC7293793; doi:10.1186/s12933-020-01052-1)
Supplement: Supplementary file 1 — Additional file 1: Table S1. Search terms used in the systematic search. Table S2. Methods and cut-off points that were used to define abnormal values of cardiometabolic risk factors. [file 12933_2020_1052_MOESM1_ESM.docx]

**Additional file**

| **Table S1. Search terms used in the systematic search.** | | |
| --- | --- | --- |
| “Severe obesity”[tiab] OR “Severely obese”[tiab] OR “Severe obese”[tiab] | Severe obesity | Severe Obesity |
| “Morbid obesity”[tiab] OR “Morbidly obese”[tiab] OR “Morbid obese”[tiab] OR "Obesity, morbid"[mesh] | Morbid obesity |  |
| “Class 3 obesity”[tiab] OR “Class 3 obese”[tiab] OR “Obesity class 3”[tiab] OR “Grade 3 obesity”[tiab] | Class 3 obesity |  |
| “Class 2 obesity”[tiab] OR “Class 2 obese”[tiab]OR “Obesity class 2”[tiab] OR “Grade 2 obesity”[tiab] | Class 2 obesity |  |
| child*[tiab] |  | Children |
| teen*[tiab] |  |  |
| adolescen*[tiab] |  |  |
| youth*[tiab] |  |  |
| young[tiab] |  |  |
| pediat*[tiab] |  |  |
| toddler*[tiab] |  |  |
| infant*[tiab] |  |  |
| “Diabetes mellitus”[mesh] OR diabetes[tiab] | Diabetes | Risk factors and outcomes |
| dysglycemia[tiab] | Dysglycemia |  |
| “Non-alcoholic Fatty Liver Disease”[mesh] OR NAFLD[tiab] OR “Non-alcoholic Fatty Liver Disease”[tiab] OR “Non alcoholic Fatty Liver Disease”[tiab] OR NASH[tiab] OR “Non-alcoholic steatohepatitis”[tiab] OR “Non alcoholic steatohepatitis”[tiab] OR “fatty liver”[tiab] OR “steatohepatitis”[tiab] | NASH |  |
| “Hypertension”[mesh] OR “hypertension”[tiab] OR “hypertensive”[tiab] OR “Hypertensive heart disease”[tiab] OR “Secondary hypertension”[tiab] OR “Hypertensive crisis”[tiab] | Hypertension |  |
| “Myocardial infarction”[mesh] OR “myocardial infarction”[tiab] OR “acute coronary syndrome”[mesh] OR “acute coronary syndrome”[tiab] OR “coronary artery disease”[mesh] OR “coronary artery disease”[tiab] OR “myocardial ischemia”[mesh] OR “ischemic heart disease”[tiab] | Acute coronary syndrome |  |
| “Stroke”[mesh] OR “stroke”[tiab] OR “cerebrovascular accident”[tiab] OR “CVA”[tiab] OR “transient ischemic attack”[tiab] OR “TIA”[tiab] | Stroke |  |
| “Heart failure”[mesh] OR “heart failure”[tiab] | Heart failure |  |
| “cardiovascular diseases”[mesh] OR “cardiovascular” | Cardiovascular |  |
| “Cardiometabolic”[tiab] OR “ | Cardiometabolic |  |
| “Mortality”[mesh] OR “mortality”[tiab] | Mortality |  |
| “Atherosclerosis”[mesh][tiab] OR “Aortic aneurysm”[mesh][tiab] OR “Aneurysm dissecting”[mesh] OR“Aortic dissecting”[tiab] OR “Peripheral vascular disease”[mesh][tiab] OR “Arterial embolism”[tiab] OR “Atheroembolism”[tiab] OR “aortitis”[mesh][tiab] | Diseases of the arteries  Atherosclerosis  Aneurysm  Dissection  Pvd |  |
| “Total cholesterol”[tiab] | Total cholesterol |  |
| “HDL”[tiab] OR “high-density lipoprotein cholesterol”[tiab] OR “high density lipoprotein cholesterol”[tiab] | HDL |  |
| “LDL”[tiab] OR “low-density lipoprotein cholesterol”[tiab] OR “low density lipoprotein cholesterol”[tiab] | LDL |  |
| “triglycerides”[tiab] | Triglycerides |  |
| “Blood pressure”[mesh] OR “blood pressure”[tiab] | Blood pressure |  |
| “Glycated hemoglobin A”[mesh] OR “glycated hemoglobin”[tiab] OR “HBA1C”[tiab] | HbA1C |  |
| “Fasting glucose”[tiab] OR “fasting plasma glucose”[tiab] | Fasting glucose |  |
| “Cardiomyopathy”[mesh][tiab] OR “Arrhythmias, cardiac”[mesh] OR “Arrhythmia”[tiab] OR “Atrial fibrillation”[mesh] OR “Atrial fibrilliation”[tiab] OR “Atrial flutter”[mesh] [tiab] OR “Pericarditis”[mesh][tiab] OR “Endocarditis”[mesh][tiab] OR “Myocarditis”[mesh][tiab] OR “Tachycardia, paroxysmal”[mesh] OR “Paroxysmal tachycardia”[tiab] OR “Aortic valve stenosis”[mesh] OR “Aortic stenosis”[tiab] OR “Aortic valve insufficency”[mesh] OR “Aortic regurgiatation”[tiab] OR “Mitral stenosis”[tiab] OR “Mitral valve stenosis”[mesh] OR “Mitral valve insufficency”[mesh] OR “Mitral regurgiation”[tiab] OR “Pulmonary embolism”[mesh][tiab] OR “Venous thromboembolism”[mesh][tiab] OR “Deep venous thrombosis”[tiab] OR Venous thrombosis[mesh] | Others:  Cardiomyopathy  Arrhythmia  Atrial fibrillation  Pericarditis  Endocarditis  Myocarditis  Paroxysmal Tachycardia  Aortic stenosis  Aortic regurgitation  Mitral stenosis  Mitral regurgitation  Venous thromboembolism |  |
| “Kidney failure, chronic”[mesh] OR “End stage renal disease”[tiab] OR “Chronic renal failure”[tiab] OR “Chronic kidney injury”[tiab] OR “Chronic kidney disease”[tiab] OR “Chronic renal disease”[tiab] OR “ESRD”[tiab] OR “CKD”[tiab] | Kidney failure |  |
| "Neoplasms"[mesh][tiab] OR "Malignancy"[tiab] OR "Cancer"[tiab] | Cancer |  |

Listed are terms used in the following combinations: severe obesity terms AND children terms and risk factors and outcomes terms. The search was limited to studies on humans and written in English. The outcomes listed are in accordance with the classification of cardiovascular disease of the American Heart Association guidelines.

| **Table S2. Methods and cut-off points that were used to define abnormal values of cardiometabolic risk factors.** | | | | | | | | |
| --- | --- | --- | --- | --- | --- | --- | --- | --- |
| **Article** | **Cardiometabolic Risk Factors** | | | | | | | |
|  | **Total Cholesterol**  **(mg/dl)** | **LDL (mg/dl)** | **HDL (mg/dl)** | **Triglycerides (mg/dl)** | **Systolic BP (mmHg)** | **Diastolic BP (mmHg)** | **Fasting Glucose (mg/dl)** | **HbA1c (%)** |
| Zhang et al, 2017 |  |  |  |  | SBP OR DBP ≥95th percentile for age and sex. | |  |  |
| Skinner et al, 2015 | ≥200 | ≥130 | <35 | ≥150 | ≥95th percentile | ≥95th percentile | ≥100 | >5.7% |
| Propst et al, 2015 |  |  | ≤45 |  | SBP OR DBP > 95th percentile for age, sex, height. | |  |  |
| Marcus et al, 2014 | ≥200 | ≥130 | ≤40 | ≥130 | ≥90th percentile OR ≥120/80 | | ≥100 |  |
| Rank et al, 2013 |  |  | <40 | ≥150 | ≥130 mmHg OR ≥85 mmHg OR treatment | | ≥100 OR treatment |  |
| Calcaterra et al, 2008 | Metabolic syndrome (>3 out of 5): 1. BMI>97^th^ percentile 2. Triglycerides>95^th^ percentile 3.HDL<5^th^ percentile 4. SBP OR DBP>95^th^ percentile 5. Impaired glucose tolerance | | | | | | | |
| Boyd et al, 2005 | ≥200 | ≥130 | <40 | ≥150 |  |  |  |  |
